# The nutrition for sport knowledge questionnaire (NSKQ): development and validation using classical test theory and Rasch analysis

**DOI:** 10.1186/s12970-017-0182-y

**Published:** 2017-08-03

**Authors:** Gina Louise Trakman, Adrienne Forsyth, Russell Hoye, Regina Belski

**Affiliations:** 1 Department of Rehabilitation, Nutrition and Sport, School of Allied Health, College of Science, Health and Engineering, La Trobe University, Melbourne, 3086 Australia; 2 Department of Management and Marketing, La Trobe Business School, College of Arts, Social Sciences and Commerce, La Trobe University, Melbourne, 3086 Australia; 3 Department of Health Professions, School of Health Sciences, Faculty of Health, Arts and Design, Swinburne University of Technology, Hawthorn, 3122 Australia

**Keywords:** Nutrition knowledge, Sports nutrition, Questionnaire, Measure, Valid, Classical test theory, Rasch analysis

## Abstract

**Background:**

Appropriate dietary intake can have a significant influence on athletic performance. There is a growing consensus on sports nutrition and professionals working with athletes often provide dietary education. However, due to the limitations of existing sports nutrition knowledge questionnaires, previous reports of athletes’ nutrition knowledge may be inaccurate.

**Methods:**

An updated questionnaire has been developed based on a recent review of sports nutrition guidelines. The tool has been validated using a robust methodology that incorporates relevant techniques from classical test theory (CTT) and Item response theory (IRT), namely, Rasch analysis.

**Results:**

The final questionnaire has 89 questions and six sub-sections (weight management, macronutrients, micronutrients, sports nutrition, supplements, and alcohol). The content and face validity of the tool have been confirmed based on feedback from expert sports dietitians and university sports students, respectively. The internal reliability of the questionnaire as a whole is high (KR = 0.88), and most sub-sections achieved an acceptable internal reliability. Construct validity has been confirmed, with an independent T-test revealing a significant (*p* < 0.001) difference in knowledge scores of nutrition (64 ± 16%) and non-nutrition students (51 ± 19%). Test-retest reliability has been assured, with a strong correlation (*r* = 0.92, *p* < 0.001) between individuals’ scores on two attempts of the test, 10 days to 2 weeks apart. Three of the sub-sections fit the Rasch Unidimensional Model.

**Conclusions:**

The final version of the questionnaire represents a significant improvement over previous tools. Each nutrition sub-section is unidimensional, and therefore researchers and practitioners can use these individually, as required. Use of the questionnaire will allow researchers to draw conclusions about the effectiveness of nutrition education programs, and differences in knowledge across athletes of varying ages, genders, and athletic calibres.

**Electronic supplementary material:**

The online version of this article (doi:10.1186/s12970-017-0182-y) contains supplementary material, which is available to authorized users.

## Background

Appropriate dietary intake can improve athletic performance, enhance adaptations to training and augment recovery from exercise [1, 2]. However, athletes have been known to consume diets that do not to meet their energy and nutrient needs [3], and a mismatch between contemporary expert recommendations and athletes’ dietary practices have previously been demonstrated [4]. Nutrition education programs improve nutrition knowledge [5–7] and higher levels of knowledge are correlated with better diet quality [7–9]. Accordingly, professionals working with sports people often provide nutrition advice [10]. Parks et al. [11] reported that the number of dietitians employed by collegiate athletic departments has quadrupled since 2010. However, globally there is limited information regarding athletes’ access to relevant and appropriate nutrition advice; ostensibly, this may vary according to the level of professionalism of their respective sport and their immediate support network. Hamilton et al. [12] reported that elite athletes in New Zealand had higher levels of knowledge than non-elite athletes. In contrast, Andrews et al. [13] found no differences between sub-elite and elite Australian soccer players. Trakman et al. [14] conducted a systematic literature review on nutrition knowledge of athletes and coaches and reported a possible relationship between athletic calibre and knowledge. However, the authors concluded that due to the heterogeneity and poor quality of Nutrition Knowledge Questionnaires (NKQ’s), athletes’ nutrition knowledge (and the factors that influence this) are difficult to ascertain [0–22]. The poor quality of NKQs is also likely to influence researchers’ ability to accurately quantify the correlation between knowledge and dietary intake [8, 15] and impact practitioners’ ability to evaluate nutrition education programs.

Trakman et al. [14] noted that a key factor affecting the quality of NKQs was a lack of adequate validation. The maximum validation score of a sports nutrition knowledge questionnaire (SNKQ) used with athletes was three out of six. More recently, Furber et al. [16] developed an SNKQ for British track and field athletes undertaking four of the six recommended validation methods; face validity testing and item analysis were not performed. Of note, the rating system used by Trakman et al. [14] was based solely on classical test theory (CTT). The CTT framework focuses on the questionnaire as a whole. It is based on correlations and assumes that all questions are equal indicators of an individual’s nutrition knowledge [17]. A key aspect of CTT is the use of the Cα statistic to measure internal reliability; however, Cα is only suitable for scales with 20 or fewer items and is frequently incorrectly used on much longer questionnaires [18]. Moreover, it is not an inherent property of a questionnaire and needs to be re-assessed each time a new sample completes the tool [18].

An alternative to CTT is Rasch analysis. Rasch analysis is a technique that was first developed in education, has been utilised to develop psychological assessment tools [19] and health related patient-reported outcomes (HR-PRO) [12, 20], and more recently has been utilised to validate questionnaires that assess knowledge of the energy content of meals and balanced meals [21, 22]. Rasch analysis offers several advantages over CTT; it allows shorter scales with multiple response formats to be developed, and because it does not rely on measures of central tendency, it is said to be more ‘stable’ across varying populations [23]. The aim of Rasch analysis is to create a unidimensional (i.e. assessing one concept) questionnaire. During Rasch analysis it is necessary to test that the questionnaire concurs with the assumptions that (1) difficult items are less likely to be answered correctly, and (2) individuals with higher levels of knowledge are more likely to answer questions correctly. These expectations are tested by assessing a range of statistics which provide feedback on: the differences between observed and expected responses; whether the difficulty of items is consistent across participants (i.e. whether items are good at discriminating between well-scoring and poor-scoring respondents); and whether items are answered consistently on the basis of participant characteristics, such as age and gender. The present study will use a novel method that evaluates items based both on CTT and Rasch analysis. To our knowledge, no SNKQ has been validated using Rasch analysis.

In addition to issues pertaining to validation, many existing SNKQs have problems with their actual content. While 13 (out of 36) studies in the review by Trakman et al. [14] covered 75% of the nutrition sub-sections that were deemed relevant, the comprehensiveness assessment was limited because the researchers did not assess the extent to which each topic was assessed or the quality of individual items. Indeed, many items appear to test out-dated dietary recommendations that are not in line with recently published guidelines such as the American College of Sports Medicine (ACSM), the International Olympic Committee (IOC), and the International Society for Sports Nutrition (ISSN) review on Sport Nutrition [24] and the multiple ISSN, IOC review papers and consensus statements on nutrition and athletic performance [1, 2, 25–33]. As above, current guidelines expound that carefully choosing the amount, type and timing of foods and fluids will optimise an athlete’s adaptations to training, performance outcomes, and recovery from exercise. They emphasise the importance of individualising nutrition, especially with regards to carbohydrate intake and hydration, and acknowledge that some supplements (e.g. creatine, caffeine, and bicarbonate) can enhance athletes’ performance, but encourage a prudent approach to supplementation [1, 2, 25, 26, 34]. The present study has based questions on these recommendations.

Further to the issues pertaining to the quality and content of existing SNKQ’s, many tools have limited cultural applicability and/or focus on measuring the knowledge of a single sport. This limits the ability of tools to be used to compare knowledge of athletes from different countries and knowledge of athletes between sports.

The aim of this study was to address the deficiencies in existing SNKQ’s by developing a new SNKQ that:

1. Has been validated using a robust methodology that includes both CTT techniques and Rasch analysis
2. Assesses knowledge of current consensus recommendations on sports nutrition
3. Assesses knowledge of all relevant aspects of sports nutrition and is generalizable to multiple sports
4. Is likely to be understood by individuals from various cultural backgrounds

It was hypothesized that the questionnaire would represent a significant improvement on currently available measures. From a research perspective, a high-quality nutrition knowledge measure will allow for more accurate assessment of factors that influence knowledge and a more a more reliable assessment of the impact of nutrition knowledge on diet quality. Moreover, for individuals working with athletes, a quality measure is likely to have practical implications, allowing for the evaluation of nutrition education programs and therefore development of more targeted education strategies that are based on gaps in knowledge.

## Methods

### Ethics approval and consent to participate

The research was approved (S16/267) by the La Trobe University’s SHE College Human Ethics Sub-Committee (SHE CHESC). Participants in the expert panel review and think-out-loud focus groups read the Participant Information Statement and signed the Consent Form. Participants who were involved in steps six to eight read the Participant Information Statement and provided consent electronically.

### Novel eight-step validation process

A novel eight-step validation method for the development of a nutrition knowledge questionnaire was designed based on an extensive review of the literature and used to validate this questionnaire [35]. The steps include: (1) Definition of Sports Nutrition Knowledge (2) Generation of items to represent sports nutrition knowledge (3) Choice of scoring system (4) Assessment of content validity by panel of experts (5) Assessment of face validity by student athletes (6) CTT analysis: Removal of items on the basis of item difficulty, item discrimination and distractor utility (7) Rasch analysis: Assessment of dimensionality and removal of item on the basis of not meeting assumptions that difficult questions are less likely to be answered correctly, and well-scoring participants are more likely to answer individual items correctly (8) Assessment of construct validity by comparing nutrition and non-nutrition students; assessment of test-retest reliability (consistency over time) by assessing correlation of test on two attempts; and re-checking of steps six and seven. The steps that make this methodology novel are the quantitative assessment of content validity, the assessment of distractor utility (how feasible incorrect multiple choice options are) and the inclusion of Rasch analysis.

**Fig. 1:**
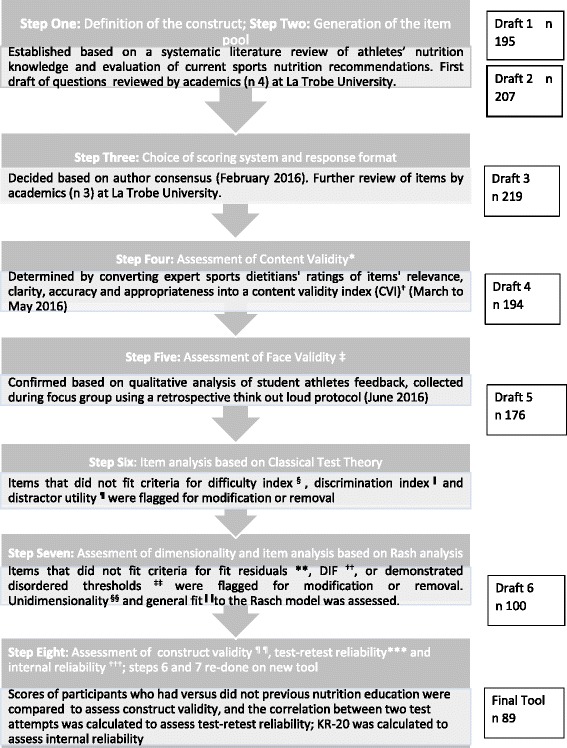
Flow chart of 8-step methadology used to develop and validate the Nutrition for Sport Questionnaire (UNSQ). * Content Validity = the measure covers all relevant topics related to sports nutrition. † CVI = Number of experts who rated an item ‘very relevant’ or ‘relevant’ divided by total number of experts; > 0.78 is adequate. ‡ Face Validity = the measure, on face value is an adequate reflection of sports nutrition. § Difficulty index = frequency with which items were answered correctly; <20% = too hard; >80% = too easy. ǁ Discrimination index = average score of top 10% of participants minus average score of bottom 10% of participants; > 0.3 is adequate. ¶ Distractor utility = frequency with which each multi-choice option is selected; > 5% = effective distractor. **Fit residuals between −2.5 and 2.5 indicate observed = expected responses. ††DIF assessed using ANOVA; non-significant *p*-value = no differences in response pattern based on participant characteristics; ‡‡ Disordered thresholds are assessed graphically. §§ Perc5% statistic <5% = scale is unidimensional (assessing one concept). ǁ ǁ SD of 0 and Mean of 1for the overall item/person interaction = perfect fit to Rasch model; a SD > 1.5 = misfit. ¶¶ Significant differences in known-group comparison scores = construct validity (questionnaire test what it is supposed to). *** Pearson’s *r* > 0.7 = test-retest reliability (stability overtime). ^†††^ KR-20 > 0.7 = Internal reliability (consistency in items)

Figure 1 provides a summary of the methods and results.

### Recruitment

Experts (step four) were recruited using purposive sampling between April and May 2016 and student-athletes (step five) were recruited using convenience sampling in June 2016. For steps six and seven, Australian Football League (AFL) Victoria community football players, La Trobe University student-athletes and other recreational athletes were invited to complete the questionnaire via email, Facebook groups, and online athlete forums. Data collection occurred between July 2016 and October 2016. For step eight, La Trobe University undergraduate and postgraduate business, health science and nutrition students were recruited via email and their Learning Management System (LMS) notice boards and recreational athletes were invited to participate via email and their team Facebook pages. Data collection took place from November 2016 until January 2017.

### Sample size calculations

To calculate a CVI (step four) three to 10 experts are needed [36]. The ideal number of participants for focus groups (step five) are six to 10 [37]. Parmenter et al. [38] recommend that to carry out CTT analysis at least one more person than the number of items are required. Pallant [23] recommends that to carry out Rasch analysis, 240 participants are ideal. At steps 6 and 7, the questionnaire had 178 items; a target of 200 participants was set to account for both the CTT and Rasch estimates. For step eight, a power analysis for an independent sample t-test was conducted in G-POWER to determine a sufficient sample size using an alpha of 0.05, a power of 0.80, a large effect size (d = 0.8), and two tails. Based on these assumptions, the desired sample size for each group (nutrition versus non-nutrition students) was 51 [39, 40].

### Statistical analysis

Missing values were assessed for normality using Kolmogorov-Smirnov statistic and compared across participant characteristics using independent t-test and ANOVA or Mann–Whitney and Kruskal-Walis, as appropriate (see Additional file 1: Supplementary Material S1). Total and sub-section scores were assessed for normality using Kolmogorov-Smirnov statistic. Parametric tests (independent t-test; ANOVA) were used for normal data. Where assumptions of normality were violated, non-parametric tests (Mann–Whitney U test; Kruskal-Wallis) were used. Normal results were reported as mean ± SD, non-normal results were reported as median and IQR. For correlations, Pearson’s r was used for parametric data, and Spearman’s r was used for non-parametric data. Individuals with and without nutrition education were compared across participant characteristics using chi-square test, to account for potential confounding. Differences in scores based on age, gender, country of birth, level of education, and history of playing sports were also assessed.

## Results

### Participants

Ten experts were invited to be involved in the content validity assessment. Six sports dietitians agreed to participate and three of these returned feedback forms; two respondents were Australian and the other was Swiss. They worked in private consultancy, research and education, and industry. Eight students participated in the retrospective think-out-loud focus group. One student who was unable to attend the session met with the researcher on a separate occasion. For the item analysis based on CTT and Rasch scaling (steps six and seven), 462 athletes started the questionnaire; after excluding data with more than 11% missing values (*n* = 259) and participants who did not meet the eligibility criteria for age (*n* = 15) there were 188 usable responses (Table 1). For the assessment of construct validity, test-retest reliability and re-evaluation of CTT and Rasch parameters (step eight), 287 students and athletes started the questionnaire and there were 181 usable responses, including 28 responses from individuals who had completed the questionnaire on two occasions (Table 2).

**Table 1:** Characteristics of participants included in the first item analysis using CTT and Rasch analysis (*n* = 188)

Characteristic	Option: N (% available data)
Gender	Male: 77 (42)
	Female: 106 (58)
Age	17–25: 45 (25)
	26–35: 73 (41)
	36–45: 35 (20)
	46–55: 18 (11)
	56–65: 8 (4)
Country of Birth (COB)	Australia: 159 (86)
	New Zealand: 4 (2)
	USA: 6 (3)
	UK: 6 (3)
	Other: 10 (5)
Marital Status	Single: 95 (51)
	Married/De-facto: 85 (46)
	Divorced: 5 (3)
Highest level of education	Primary School: 2 (1)
	High School: 23 (13)
	Vocational education or other diploma: 19 (10)
	University:
	Bachelors/Undergrad degree: 90 (49)
	Honors/Master: 43 (23)
	Doctorate: 7 (4)
Main sport played	AFL: 77 (42))
	Basketball: 3 (2)
	Cricket: 19 (10)
	Cycling: 3 (2)
	Distance running: 30 (16)
	Netball: 10 (5)
	Soccer/Football: 8 (4)
	Swimming: 4 (2)
	Rowing: 2 (2)
	Other: 28 (15)
Hours Training /Week	1.0–28.0 (2.7+/−1.1)
Athletic Caliber	International: 11 (6)
	National: 16 (9)
	State: 36 (21)
	Local: 88 (50)
	Recreational: 25 (14)
Paid to play sport	Yes: 9 (5)
	No: 172 (95)
Formal nutrition studies	Yes: 35 (19)
	No: 147 (81)
Advice to change diet	Yes: 91 (49)
	No: 96 (51)

There was data missing for gender (*n* = 5), age (*n* = 9), COB (*n* = 3), marital status (*n* = 3), education (*n* = 4), main sport played (*n* = 4), hours training (*n* = 12), athletic caliber (*n* = 12), paid to play sport (*n* = 7), formal nutrition studies (*n* = 6), advice to change diet (*n* = 1). Percentages have been rounded to the nearest whole figure and reported based on available data

**Table 2:** Characteristics of participants included in Study Two analysis (*n* = 181)

Characteristic	First completion: n (% available data)	Second completion: n (%)
Gender	Male: 36 (26)	Male: 6 (21)
	Female: 103 (74)	Female: 22 (79)
Age	17–25: 77 (53)	17–25: 15 (54)
	26–35: 35 (24)	26–35: 5 (18)
	36 - 45: 21 (15)	37 - 45: 3 (11)
	46 - 55: 6 (4)	46 - 55: 3 (11)
	>55: 6 (4)	>55: 2 (7)
Country of Birth (COB)	Australia: 111 (80)	Australia: 23 (82)
	Outside Australia: 27 (20)	Outside Australia: 5 (18)
Marital status	Single: 95 (68)	Single: 18 (64)
	Married/De-facto: 39 (28)	Married/De-facto: 7 (25)
	Divorced: 5 (4)	Divorced: 3 (11)
Highest level of education	High school: 3 (2)	
	Vocational training or other diploma: 3 (2)	Bachelor/undergraduate degree: 18 (64)
	Bachelor/undergraduate degree: 87 (60)	Honours/master’s degree: 7 (25)
	Honours/master’s degree: 35 (24)	Doctoral degree): 3 (11)
	Doctoral degree: 16 (11)	
Nutrition education	Yes: 77 (52)	Yes: 20 (71)
	No: 70 (48)	No: 8 (29)
Plays sport on a regular basis	Yes: 88 (66)	Yes: 12 (43)
	No: 45 (34)	No: 16 (57)

For first round completion (n 153), there was data missing for gender (*n* = 14); age (*n* = 8); COB (*n* = 15); marital status (*n* = 19); education (*n* = 9); nutrition education (*n* = 6); sport (*n* = 20). There was no missing data for second round completion (n 28). Percentages have been rounded to the nearest whole figure and reported based on available data

There did not appear to be any differences between individuals who completed and did not complete the questionnaire (See Additional file 1: Supplementary Material S2).

Individuals who studied nutrition were more likely to be female, have a tertiary education and were younger (See Additional file 1: Supplementary Material S3).

### The final questionnaire

Sports nutrition knowledge was defined as *“Knowledge of concepts and processes related to nutrition for optimal athletic performance including knowledge of weight management; hydration and fuelling strategies for before, during and after training/performance; supplementation and alcohol use”*. The original test plan (see Additional file 1: Supplementary Material S3) follows logically from this. Each correct answer was awarded a point. The final questionnaire had 89 items and six unidimensional sub-sections; weight management (*n* = 13); macronutrients (*n* = 30); micronutrients (*n* = 13); sports nutrition (*n* = 13); supplements (*n* = 12) and alcohol (*n* = 8). The sports nutrition section covers hydration, the pre-completion meal, nutrition during exercise and recovery nutrition. The item response formats include agree/disagree/not sure, multiple choice, and effective/not effective/not sure. The questionnaire is designed to be administered online and includes pictures to reduce responder fatigue. The number of items at each stage of validation is represented in Fig. 1.

### Validity

The experts reported that the questionnaire covered all relevant topics and therefore content validity was confirmed (step four). Most items that were rated poorly for accuracy, relevance, appropriateness and clarity were removed (*n* = 25) although some exceptions were made. The number of items that did not meet the CVI and examples of changes made based on expert’s feedback are included as Additional file 1: Supplementary Material S4.

Student athletes were able to identify what each section was attempting to capture. Where there were issues with wording of questions, items were removed (*n* = 17) or modified (*n* = 16), so face validity was confirmed (step five). Examples of changes made based on students’ feedback are included as Additional file 1: Supplementary Material S5.

Construct validity was demonstrated (step eight) because there was a significant difference in the total scores for those who had nutrition education and those who did not have nutrition education (*P* < 0.001) (Table 3). Individuals with nutrition education also scored better across all sub-sections except alcohol (Table 3). No other participant characteristics were found to have a significant effect on scores (See Additional file 1: Supplementary Material S6).

**Table 3:** Comparison of scores between individuals with and without previous nutrition education; test-retest reliability; internal reliability analysis; results of Rasch analysis

Section	Score (%): Nutrition education	Score (%): No nutrition education	Score p-level	Pearson’s Correlation R (p- level)	KR-20^+^	Dimensionality (Per < 5%)	Bonferroni adjusted p-level	Item fit statistics
*Overall (*n* = 89)	64. 65 (16)	52 (19)	<0.001	0.92 (<0.001)	0.0.87	19.3%	0.0005	Persons SD = 1.0271, Mean = −0.0938
								Items SD = 1.324, Mean = 0.0724
								Chi-Square probability = 0.000
**WeightManagement (*n* = 13)	77 (23)	62 (31)	<0.001	0.0.81 (<0.001)	0.62	2.8%	0.004	Persons SD = 0.62, Mean = −0.12;
								Items SD = 1.06; Mean = 0.02;
								Chi Square probability = 0.01 > 0.004
*Macronutrients (*n* = 30)	78 (15)	61 (16)	<0.001	0.0.81 (<0.001)	0.78	5.0%	0.002	Persons SD = 0.72, Mean = −0.11;
								Items SD = 1.35; Mean = 0.05;
								Chi Square probability = 0.0000 < 0.002
**Micronutrients (*n* = 13)	69 (23)	50.0 (30.8)	<0.001	0.76 (<0.001)	0.71	0.0%	0.005	Persons SD = 0.80, Mean = −0.21;
		50 (31)						
								Items SD = 1.16; Mean = −0.36;
								Chi Square probability = 0.0002 < 0.0004
**Sports Nutrition (*n* = 13)	54 (23)	46 (23)	<0.001	0.0.73 (<0.001)	0.63	1.7%	0.004	Persons SD = 0.57, Mean = −0.19;
								Items SD = 1.39; Mean = 0.06;
								Chi Square probability = 0.009 > 0.004
**Supplements (*n* = 12)	33 (33)	25 (25)	<0.001	(0.60.35 (0.6)	0.0.69	0.0%	0.004	Persons SD = 0.69, Mean = −0.13;
								Items SD = 0.82; Mean = −0.20;
								Chi Square probability = 0.02 > 0.004
**Alcohol (*n* = 8)	63 (31)	63 (25)	0.0.80	0.66 (<0.001)	0.0.51	0.0%	0.007	Persons SD = 0.65, Mean = −0.14;
								Items SD = 1.1; Mean = −0.09;
								Chi Square probability = 0.003 < 0.007

*Normally distributed; scores reported as Mean (SD) **Non-normally distributed; scores reported as Median (IQR). CTT: Significant differences in groups scores indicate construct validity; Pearson’s r of 0.7 indicates adequate test-retest reliability; a KR-20 of 0.7 indicates adequate internal reliability. Rasch analysis: A non-significant Bonferroni adjusted chi-square probability indicates items are equally difficult across all participants (i.e. are good discriminators); a SD of 1 and Mean of 0 (for persons/items) indicate perfect fit to the Rasch model; a SD of >1.5 indicates the assumptions that difficult items are less likely to be answered correctly or well-scoring participants are more likely to answer questions correctly has not been met

Internal reliability of sub-sections ranged from 0.51 to 0.78 (Table 3). All sections except alcohol achieved or approached adequate values for internal reliability given the number of items in each sub-section. Test re-test reliability ranged from 0.35 to 0.81; all sections except supplementation achieved or approached adequate values for test-retest reliability (Table 3).

### Item analysis based on CTT

Item analysis using CTT was carried out at steps 7 and 8. Most items that did not meet the criteria for item difficulty, item discrimination and distractor utility were removed or modified, although some exceptions were made, and the final tool included 15 items that did not meet these criteria. Examples of changes made on the basis of item analysis are included as Additional file 1: Supplementary Materials S7 and S8.

### Fit to the rash model

Based on the Rasch analysis conducted on the tool administered at step eight, the questionnaire as a whole was multidimensional. Eleven items were removed because they were shown to be poor discriminators and/or were answered differently (demonstrated DIF) on the basis of age or gender. With these items removed, the questionnaire as a whole was still mutli-dimensional, but the six sub-sections were shown to be unidimensional (Table 3). Each sub-section also had an adequate mean, SD for the overall item/trait interaction statistics, meaning that the expectation that difficult items were more likely to be answered incorrectly and well scoring participants were more likely to answer questions correctly was met. The chi-square probability for macronutrients, micronutrients and alcohol was significant (Table 3); this occurred because some items that did not meet the Rasch indicator requirements were kept because they were deemed important to assess in an SNKQ.

## Discussion

### The questionnaire

Due to the limitations with existing SNKQs, researchers have raised concerns regarding the accuracy of previous reports of athletes’ nutrition knowledge, and have postulated that the relationship between nutrition knowledge and dietary intake may have been misjudged [8, 14, 41, 42]. The aim of this study was to create an SNKQ that tested awareness of current consensus recommendations, was adequately validated and could be used with athletes from a range of sports. The newly developed tool, the *Nutrition for Sport Knowledge Questionnaire* (NSKQ), has 89 individual items (44 questions, with some having multiple parts) covering six distinct subsections The questionnaire takes around 25 min to complete and is comparable in length to the GNKQ [40], which has 113 items and the SNKQ developed by Zinn et al. [43], which has 88 items. Since the questions are based on consensus guidelines, several items assess theoretical knowledge. Questions that assess practical knowledge have also been included. The tool is more comprehensive than existing measures as it includes an alcohol sub-section and adheres to a detailed test plan. The questions are based on current guidelines. For example, rather than ask about carbohydrate requirements as % total calorie intake, we have included a question on requirements in g/kg/day and specified the type (‘endurance’) and intensity (‘moderate to high’) of activity. Likewise, in contrast to other existing tools, our hydration question reflects findings that using thirst to judge fluid needs can maximise performance and current recommendations that hydration plans should be tailored to the individual [31, 44]. Moreover, questions on the timing of the pre-completion meal and recovery snack have intentionally been omitted. It is too difficult to assess the correct answer to these questions given the increasing evidence to support the positive benefits of periodizing nutrition based on the goals of individual sessions and overall training schedule [4, 28].

### Validity and reliability

The questionnaire has demonstrated face and content validity based on student-athletes and sports dietitians’ judgements. In contrast to previous tools, the content validity has been assessed quantitatively, using a CVI [36]. Individuals who reported undertaking studies in human nutrition achieved higher scores across all sections, except alcohol, indicating that the questionnaire has good construct validity. The group with a nutrition education were younger and were more likely to be female and tertiary educated. In contrast to previous reports, there was no significant difference in performance based on age or gender [41, 40]. Therefore, the variations in knowledge between groups are unlikely to be due to underlying differences in participant characteristics. Test-retest reliability was assessed based on the correlation between individuals’ (who repeated the questionnaire) test scores. A limitation of this method is that motivated individuals may upskill between attempts. The average total score was higher for attempt two. Nevertheless, overall test-retest reliability was high, and all sub-sections, except ‘supplements’, achieved (or approached) adequate test-retest reliability. Participants performed most poorly on the supplement section. Therefore, it is feasible that the supplement test-retest result occurred due to participants guessing answers. The overall internal reliability was very high, and the internal reliability of most sub-sections (except alcohol) reached or approached the requisite 0.7 value. As expected, there appeared to be a relationship between the number of items and KR-20. Streiner [18] recommends that KR-20 be interpreted with caution if there are more than 20 items; the overall scale and macronutrient sub-section exceeded this value.

Based on the Rasch analysis, the overall Item/person interaction statistics were adequate, indicating compliance with the expectations that difficult questions were less likely to be answered correctly, and individuals who performed well overall were more likely to answer individual questions correctly. The questionnaire as a whole was multi-dimensional, but with problematic items removed, each section was shown to be unidimensional. Therefore, sections can be used independently, as required. Where the whole tool is used, sub-sections rather than total score should be reported.

All items were written so that units and food names were generic and likely to be understood by individuals of varying cultural backgrounds; however, additional evaluation is required to confirm the functionality of the tool in groups who differ from the present cohort. The fact that country of birth did not influence scores, and the use of Rasch analysis, which produces questionnaires that are independent of the sample used for validation, give some indication that the tool is likely to also be valid in other groups.

### Limitations

A limitation of this study is that we were unable to calculate response rates because we distributed the questionnaire using Facebook groups and online athlete forums, making total exposure unclear. The completion rates for step 7 (where item analysis and Rasch analysis was undertaken) were relatively low (~45%), but there did not appear to be any relationship between participant characteristics (other than sport played, country of birth) and completion rate (S2). The completion rate for step eight (~66%) was adequate [45]. The sample size is another potential limitation. For step seven, there were 188 responses for a 176 item measure; for step eight, there were 181 responses for a 100 item measure. The target sample size for both studies was 200. However, there is some evidence to recommend that samples as small as 30–50 are appropriate for CTT [46]. Similarly, Chen et al. [47] modelled Rasch with varying sample sizes and found that stable results can be achieved with samples of around 100.

A limitation of the questionnaire itself is that the length may be prohibitive, especially for athletes balancing training and work/study who are often time-poor. In addition, some items were poor discriminators. This was reflected by low item discrimination in CTT and the significant chi-square probability of the micronutrient, macronutrient and alcohol sections. For several questions, the poor item discrimination can be explained by the item’s relatively high or low difficulty index. That is, when a question is answered correctly (or incorrectly) by a large proportion of individuals, the overall range of responses is minimal, and therefore it is hard to achieve a meaningful difference between high and low scoring individuals. Many of these items were kept because they tested important concepts, providing valuable feedback on gaps in knowledge. Item discrimination is worth re-evaluating using larger samples of predominantly athletes (not including nutrition students). Likewise, future studies may focus on creating a short-form tool that can be used for rapid assessment of nutrition knowledge. A short-form tool would be useful in research settings where the correlation between knowledge and other factors is being assessed. A short-form tool may also have utility in the elite setting as a ‘screening’ tool for professionals working with athletes, i.e. to identify individuals who need nutrition education and extra support.

At present the NSKQ has only been validated in an Australian population. Future studies could focus on validation to confirm reliability and validity in other regions.

### Strengths

A key advantage of the questionnaire is that it has been validated using a robust methodology. To our knowledge, this is one of very few NKQ to be assessed against the Rasch Model. Likewise, it is the only tool to assess content validity qualitatively and to assess distractor utility - a distractor that is too obviously wrong will significantly increase the chances of respondents guessing a correct answer; this type of analysis is valuable. Importantly, the authors have considered the limitations of the statistics and accordingly made decisions that focused on the quality of the overall tool. In addition, the questions (and their correct answers) are based on the most recent evidence and recommendations with regards to sports nutrition; they are generalizable to most sports and enable comparison across disciplines. The tool uses food terms and measurement units that are likely to be understood by athletes from a range of countries. Moreover, the tool is detailed and therefore can assess gaps in knowledge. The NSKQ has been designed to be administered online and can provide participants with immediate feedback with regards to correct answers to questions. This is likely to be especially helpful for athletes who do not have access to professional support. The online format provides unique opportunity to direct participants to reputable and relevant resources based on their outcomes.

## Conclusions

An 89-point general and sports nutrition knowledge questionnaire with six distinct sub-sections has been developed and validated using multiple relevant methods. Three (weight management, sports nutrition, supplements) of the six sub-sections fit the Rasch model. The steps the researchers have taken to ensure the tool is current and adequately validated were robust, and the questionnaire represents an improvement on available measures. Coaches, scientists and nutrition counsellors will benefit from this tool because it will allow them to target their education based on gaps in athletes’ knowledge**.** In a team sports setting, the NSKQ may also be useful as a screening tool, to identify players who require additional educational support. Widespread utilisation of the tool in the long-term will allow for more accurate evaluation of nutrition knowledge, education programs and comparisons across athletes of varying genders, ages, education levels, and calibres.

## Additional file

Additional file 1: Supplementary Material S1. Methods used for missing variables analysis. In the original design, the demographic questions were asked last; therefore, it was not possible to complete a full assessment of factors that led participants to drop-out. Using available data, missing values (%) were compared across several participant characteristics. The continuous variables age, hours training per week, years playing sport, and country of birth were transformed into categorical variables. Country of birth was transformed into a dichotomous variable (Born in Australia/Not Born in Australia). The dependent variable, missing data, was assessed for normality using the Kolmogorov-Smirnov statistic. Assumptions of normality were violated (Statistic 0.245, df 462, *p* < 0.001). Therefore non-parametric tests were used; Mann-Whitney was used dichotomous variables (gender [male/female], being born in Australia [yes/no], being paid to play sport [yes/no], previously having undertaken nutrition studies [yes/no], previously having been given advice to change diet [yes/no]) and Kruskal-Wallis was used for categorical variables with three or more response options (sport played, highest level of education, highest level sport played, years playing sport, hours training). **Supplementary Material S2.** Relationship between missing values (%) and participant characteristics. **Supplementary Material S3.** Original ‘Universal Sports Nutrition Knowledge Questionnaire’ test plan. **Supplementary Material S4.** Summary of items that did not meet the requisite 0.3 value for CVI. **Supplementary Material S5.** Examples of changes made to the questionnaire based on the student athletes’ feedback. **Supplementary Material S6.** Comparison of scores across participant demographics at step 8. **Supplementary Material S7.** Examples of changes made based on item analysis using classical test theory at step 7. **Supplementary Material S8.** Examples of changes made to questionnaire based on CTT and Rasch re-analysis (step 8). (DOCX 36 kb)

## Abbreviations

NKQ: Nutrition knowledge questionnaire
SNKQ: Sports nutrition knowledge questionnaire
