# Supplementary material for: The nutrition for sport knowledge questionnaire (NSKQ): development and validation using classical test theory and Rasch analysis
Source: J Int Soc Sports Nutr. 2017 Aug 3;14:26. doi: 10.1186/s12970-017-0182-y (PMC5543556; doi:10.1186/s12970-017-0182-y)
Supplement: Supplementary file 1 — Methods used for missing variables analysis. In the original design, the demographic questions were asked last; therefore, it was not possible to complete a full assessment of factors that led participants to drop-out. Using available data, missing values (%) were compared across several participant characteristics. The continuous variables age, hours training per week, years playing sport, and country of birth were transformed into categorical variables. Country of birth was transformed into a dichotomous variable (Born in Australia/Not Born in Australia). The dependent variable, missing data, was assessed for normality using the Kolmogorov-Smirnov statistic. Assumptions of normality were violated (Statistic 0.245, df 462, p < 0.001). Therefore non-parametric tests were used; Mann-Whitney was used dichotomous variables (gender [male/female], being born in Australia [yes/no], being paid to play sport [yes/no], previously having undertaken nutrition studies [yes/no], previously having been given advice to change diet [yes/no]) and Kruskal-Wallis was used for categorical variables with three or more response options (sport played, highest level of education, highest level sport played, years playing sport, hours training). Supplementary Material S2. Relationship between missing values (%) and participant characteristics. Supplementary Material S3. Original ‘Universal Sports Nutrition Knowledge Questionnaire’ test plan. Supplementary Material S4. Summary of items that did not meet the requisite 0.3 value for CVI. Supplementary Material S5. Examples of changes made to the questionnaire based on the student athletes’ feedback. Supplementary Material S6. Comparison of scores across participant demographics at step 8. Supplementary Material S7. Examples of changes made based on item analysis using classical test theory at step 7. Supplementary Material S8. Examples of changes made to questionnaire based on CTT and Rasch re-analysis (step 8). (DOCX 36 kb) [file 12970_2017_182_MOESM1_ESM.docx]

**S1** Methods used for missing variables analysis

In the original design, the demographic questions were asked last; therefore, it was not possible to complete a full assessment of factors that led participants to drop-out. Using available data, missing values (%) were compared across several participant characteristics. The continuous variables age, hours training per week, years playing sport, and country of birth were transformed into categorical variables. Country of birth was transformed into a dichotomous variable (Born in Australia/Not Born in Australia). The dependent variable, missing data, was assessed for normality using the Kolmogorov-Smirnov statistic. Assumptions of normality were violated (Statistic 0.245, df 462, p <0.001). Therefore non-parametric tests were used; Mann-Whitney was used dichotomous variables (gender [male/female], being born in Australia [yes/no], being paid to play sport [yes/no], previously having undertaken nutrition studies [yes/no], previously having been given advice to change diet [yes/no]) and Kruskal-Wallis was used for categorical variables with three or more response options (sport played, highest level of education, highest level sport played, years playing sport, hours training).

**S2** Relationship between missing values (%) and participant characteristics.

| Participant Characteristic (available %) | Missing Values (Sample Size): Median % Missing value (Interquartile Range) | P-level |
| --- | --- | --- |
| Gender (72.08) | Male (164): 12.35 (83.1)  Female (169): 6.0 (57.8) | 0.299 |
| Age (69.25) | < 17 (15): 10.80 (74.7)  17 – 25 (109): 9.0 (81.9)  26 – 35 (101): 5.4 (62.7)  46 -55 (28): 79.0(83.1)  56 – 65 (13): 7.8 (81.3)  >65 (1): 18.1 (84.3) | 0.240 |
| Country of Birth (72.73) | Australia (267): 6.0 (63.3)  Not Australia (69): 60.2 (88.6) | 0.003 |
| Marital Status (80.24) | Single (189): 7.2 (82.5)  Married/De-facto (14): 6.6 (77.9)  Divorced (8): 5.4 (88.7) | 0.789 |
| Highest level of education (72.73) | Primary School (2): 6.3 (n/a)  High School (current) (36): 13.3 (79.7)  High School (completed) (37): 7.2(83.1)  Vocational education or other diploma (35): 7.20 (81.3)  Bachelors (152): 6.6 (82.5)  Honors/Master (62): 4.8 (58.4)  Doctorate (12): 11.4 (84.5) | 0.414 |
| Main sport played (73.16) | AFL (90): 5.7 (5.0)  Basketball (25): 6.6 (16.0)  Cricket (35): 5.4 (82.5)  Cycling (6): 26.80 (86.6)  Distance running (46): 3.6 (84.3)  Hockey (4): 79.0 (29.0)  Netball (25): 65.1 (83.8)  Soccer/Football (13): 4.2 (54.5)  Swimming (8): 35.0 (86.3)  Rowing (2): 3.3 (6.6)  Rugby: 88.9 (31.4)  Other (76): 65.4 (87.8) | <0.001 |
| Hours Training /Week (69.48) | 1 -2 (34): 6.0 (62.1)  3 -5 (95): 6.6 (81.9)  6 -8 (83): 4.8 (83.7)  9 -10 (58): 6.6 (82.1)  >10 (39): 12.0 (77.1) | 0.657 |
| Highest level sport played at (69.70) | International (21): 7.2 (87.35)  National (51): 10.2 (75.9)  State (67): 6.6 (83.7)  Local (131): 6.0 (61.5)  Recreational (52): 9.6 (83.7) | 0.856 |
| Years play sport (62.83) | 1 – 5 (93): 6.0 (63.0)  6 -10 (91): 6.6 (83.1)  11 – 15 (43): 6.0 (82.5)  >15 (94): 53.00 (91.6) | 0.902 |
| Paid to play sport (69.70) | Yes (21): 60.2(82.8)  No (301): 6.0 (79.2) | 0.264 |
| Formal nutrition studies (64.38) | Yes (56): 5.4 (76.8)  No (264): 6.6 (73.5) | 0.894 |
| Advice to change diet (68.83) | Yes (162): 8.40 (82.5)  No (156): 6.60 (63.2) | 0.581 |

**S3** Comparison of participants who had studied nutrition versus had not studied nutrition

|  | Previous History of Formal Nutrition Education: [n (%)] | No Previous History of Formal Nutrition Education: [n (%)] | p-level^+^ |
| --- | --- | --- | --- |
| *Age | 17 -25: 61 (64.9)  26 – 35: 18 (19.1)  36 plus: 15 (16.0) | 17 -25: 25 (34.2)  26 – 35: 22 (30.1)  36 plus: 26 (35.6) | 0.000 |
| Gender | Male: 17 (18.1)  Female: 77 (81.9) | Male: 25 (34.2)  Female: 48 (65.8) | 0.027 |
| Born in Australia | Yes: 80 (85.1)  No: 14 (14.9) | Yes: 54 (75.0)  No: 18 (25.0) | 0.151 |
| *Tertiary Education | Yes: 95 (100%) | Yes: 71 (92.2)  No: 6 (7.8) | 0.007 |
| Involvement in sport | Yes: 59 (62.8)  No: 35 (37.2) | Yes: 41 (61.2)  No: 26 (38.8) | 0.970 |
| N = 181 (first and second round completion for step 8); there was data missing for gender (n=14); age (n =6); COB (n=15); marital status (n =19); education (n=9); nutrition education (n=6); sport (n=20). There was no missing data for second round completion. *Regrouped to ensure at least five participants in each category; ^+^ p-level was determined using continuity correction | | | |

**S3** Original ‘Universal Sports Nutrition Knowledge Questionnaire’ test plan

| Nutrition Sub-Topic | Key Aspects |
| --- | --- |
| Label reading*  Energy, weight management | Energy density |
|  | Energy balance |
|  | Energy availability+ |
|  | Safety of weight loss diets |
|  | Weight loss myths |
|  | Aetiology of obesity+ |
| Carbohydrates, Fats, Proteins | Role |
|  | Recommendations |
|  | Quality |
|  | Sources |
| Micronutrients; especially B-group, Iron, Zinc, Magnesium, Calcium, | Role |
|  | Recommendations |
|  | Sources |
| Hydration | Consequences of dehydration+ |
|  | Monitoring hydration |
|  | Fluid recommendations (amount and timing) |
|  | Fluid recommendations (type) |
| Pre-Exercise Nutrition | Carbohydrate loading |
|  | Components of the pre-competition meal |
| Nutrition During Exercise | Fuel needs for skeletal muscle |
|  | Components of food eaten during exercise |
| Recovery | Importance of recovery |
|  | Components of the recovery meal |
| Supplements | Safety and legality |
|  | Efficacy  Role of individual supplements |
| Alcohol^+^ | Standard drinks  Safe alcohol consumption (long-term risk)  Safe alcohol consumption (short term risk)  Alcohol and recovery |
| *removed after study one; ^+^ added after group internal review | |

**S4** Summary of items that did not meet the requisite 0.3 value for CVI

| Domain | Items that did not meet rating [N (%)] |
| --- | --- |
| Relevance | 74 (33.8) |
| Appropriateness | 77 (35.2) |
| Clarity | 56 (25.6) |
| Accuracy | 61 (27.9) |
| Two – three domains | 51 (23.3) |
| All domains | 27 (12.3) |

| Question/Response option | Decision | Justification |
| --- | --- | --- |
| Which meal do you think is best lunch for an athlete trying to lose weight? Assume they are eating an appropriate breakfast and dinner. Correct option: A mixed meal that includes a small serving of brown rice, chicken breast, and  plenty of greens (e.g. broccoli) | Modify. Change correct option to: A mixed meal that includes a small-moderate serving of meat and carbohydrate (e.g. small bowl pasta with lean mincemeat and vegetable sauce) plus a large side salad | Reviewer three comment: “Promoting the idea of chicken and broccoli is good. Very big in the fitness industry and becoming an unhealthy addiction. What about a small bowl of spag bol with lean mince + a big side salad” |
| How much protein do you think experts recommend athletes should have after completing a resistance exercise session? Correct option: 15 – 25 g | Modify. Change correct option to 0.3g/kg body weight/day (15 – 25 g for most athletes). | Reviewer one comment: “For a 110kg rugby player this is not enough protein. Recommendations are now per kg body weight so 0.3g/kg body mass” |
| “When is carbohydrate loading likely to increase performance?” (a) In events lasting longer than 90 minutes only(b) In events lasting longer than 2.5 hours only (c) In team sports such as football (d) In all of the scenarios described above€ never (f) not sure) | Deleted | LOW CVI for accuracy, relevance, clarity and appropriateness |

**S5** Examples of changes made to the questionnaire based on the student athletes’ feedback

| Question/Response option | Decision | Justification |
| --- | --- | --- |
| The following are some strategies one might try to lose weight. Please indicate if you think these are effective, not effective, or you are not sure. 1. Increase intake of bulky low-energy foods such as vegetables | Modify. Remove ‘Bulky”. | Participant one: “Bulky sounds like the food is high in calories”  Participant five: “Yeah, Bulky makes me think of foods that are heavy and rich” |
| All athletes should take antioxidant supplements because exercise increases the production of free radicals | Modify. Change statement to: All athletes should take antioxidant supplements | Five participants were unfamiliar with the term ‘Free Radicals”; statement is double barrelled |
| Carbohydrates can impair mental performance and cause brain fog commonly referred to as 'grain brain' | Delete | All participants had not heard the terms Brain Fog and ‘Grain Brain’ |

**S6** Comparison of scores across participant demographics at step 8

| Characteristic | Category: Score (%) | P-level |
| --- | --- | --- |
| Gender | Male: 60.7  Female: 58.6 | 0.41 |
| Age | 17 to 25: 58.8  26 to 35: 61.5  36 plus: 56.25 | 0.58 |
| Country of Birth | Australia: 59.3  Outside Australia: 58.7 | 0.85 |
| Level of education | High School: 41.6  Vocational education or other diploma: 43.8  Bachelor: 58.4  Honors/Masters: 59.4  PhD: 64.6 | 0.12 |
| Plays Sport | Yes: 59.24  No: 58.4 | 0.91 |

**S7** Examples of changes made based on item analysis using classical test theory at step 7

| Question | Decision | Justification |
| --- | --- | --- |
| Which of the following do you think is an example of a "Standard Drink"? (a) 30 - 45 ml/1 - 1.5 fluid ounces of pure spirits (b) One half of a bottle (350 ml/ 12 fluid ounces) of red wine (c) A pint (425 ml/ 14 fluid ounces) of full strength beer (d) Not Sure | Modify. Change incorrect option to: “One quarter of a bottle of wine (175mL/6 fluid ounces) of red wine” | ½ bottle of red wine chosen by only 1.1% of participants. |
| Do you think these foods are low or high in carbohydrate- cheese | Delete | Negative discrimination index; answered correctly by ~85% of participants |
| Please select high or low for each nutrient, to create a description of the type of meal that you think is best for a recovery meal/snack. Fluid; Carbohydrate; Protein; Salt | Modify. Regarding foods eaten after competition, athletes should aim to consume foods that are high in: Fluid and Protein/ Fluid, carbohydrate, protein and salt/ Fluid, carbohydrate and protein/ Not Sure | Fluid answered correctly by 95.2%; Protein answered correctly by 85.6%. All options except carbohydrate had low discrimination index (0.06 – 0.11) |

**S9** Examples of changes made to questionnaire based on CTT and Rasch re-analysis (step 8)

| Question | Decision | Justification |
| --- | --- | --- |
| When do you think athletes should consume a post-exercise recover snack? (a) Within 2 hours of finishing exercise (b) It depends on the time until their next important training or performance session (c) No snack is needed, rather athletes should wait until their next scheduled meal to eat (d) Not Sure | Delete | Total glycogen repletion will be achieved so long as adequate carbohydrate is consumed; and timing and spread of protein appears to me most important. However, this concept appears too nuanced to test. Especially considering that a very common recommendation is to eat within 30 minutes of finishing exercise. Thus the decision was made not to test this at this time. “C” was a poor distractor; 94% answered incorrectly. |
| Which of the following DOES NOT impact how much should be eaten following a training session (a) The intensity of the session (b) Your hunger levels (c) The goals of the session (d) Your Weight goals € Not Sure | Delete | High fit residual; poor discriminator (CTT & IT). “A” poor distractor. |
| Polyunsaturated fats are mainly found in?(a) Vegetable oil (b) Eggs (c) Dairy foods (d) Almonds and peanuts (e) Not Sure | Delete | All other question on types of fat had been removed. This question is potentially controversial as all food contain a % of polyunsaturated fats; and some vegetable oils are high in trans fats. “B” was a poor distractor. Poor discriminator (IRT). |
| Which of the following supplements do you think is banned by the World anti-doping agency (WADA)? (a) Glycerol (b) Bicarbonate (c) Carnitine (d) b-Hydroxy-methyl butyrate (HBM) | Minor modification. Changed (d) to Caffeine, which was previously banned. | Likely (C) thrown people of as sounds long and ‘scientific’. B poor distractor. 94.9% answered incorrectly. Poor discriminator (CTT). |
| For general health, how many alcohol free days per week do you think experts recommend we should have? (a) At least 2 (b) At least 4 (c) Alcohol should be completely avoided | Major modification. *For individuals who choose to drink alcohol, to reduce the risk of alcohol-related harm over a lifetime, no more than [ ] standard drinks should be consumed per day: (1) (2) (3) (4)* | NHMRC no longer recommends alcohol free days, rather suggests zero to two drinks per day. High fit residual. Poor discriminator (both). |
